# Elucidating the putative role of cannabigerol: a hypothesis-generating review of neuroinflammatory and neuroprotective mechanisms with implications for drug-resistant epilepsy

**DOI:** 10.3389/fphar.2026.1755956

**Published:** 2026-03-16

**Authors:** Aleidy Patricio-Martínez, Soledad Hernández-Percastegui, Nadezhda Tamara Tzompantzi Juarez, Felipe Patricio, Ilhuicamina Daniel Limón

**Affiliations:** 1 Laboratorio de Neurofarmacología, Facultad de Ciencias Químicas, Benemérita Universidad Autónoma de Puebla, Puebla, Mexico; 2 Facultad de Ciencias Biológicas, Benemérita Universidad Autónoma de Puebla, Puebla, Mexico; 3 Facultad de Medicina Veterinaria y Zootecnia, Benemérita Universidad Autónoma de Puebla, Tecamachalco, Puebla, Mexico

**Keywords:** cannabidiol, cannabigerol, drug-resistant epilepsy, neurodegeneration, neuroinflammation

## Abstract

Drug-resistant epilepsy (DRE) affects approximately 30% of individuals with epilepsy and remains a major clinical challenge despite the availability of multiple antiseizure medications (ASMs). Beyond recurrent seizures, accumulating evidence implicates chronic neuroinflammation, blood–brain barrier (BBB) dysfunction, excitotoxic injury, and progressive neurodegeneration as processes associated with epileptogenesis and disease progression. While cannabidiol (CBD) has demonstrated clinical efficacy in specific DRE syndromes, increasing recognition of these mechanisms has motivated interest in exploratory, mechanism-oriented approaches that extend beyond direct seizure suppression. Cannabigerol (CBG) is a non-psychoactive phytocannabinoid with a pleiotropic pharmacological profile, interacting with cannabinoid receptors, transient receptor potential (TRP) channels, nuclear receptors such as peroxisome proliferator-activated receptor gamma (PPARγ), and additional neuromodulatory targets. Preclinical studies indicate that CBG can modulate inflammatory, oxidative, and cell-survival pathways across diverse experimental models of neuroinflammatory and neurodegenerative injury. Importantly, most available evidence derives from non-epilepsy paradigms or *in vitro* systems, and direct support for antiseizure efficacy or disease modification in epilepsy remains limited. This review synthesizes current preclinical evidence on the molecular targets and mechanistic actions of CBG, with particular emphasis on neuroimmune modulation and neuronal vulnerability, while critically addressing the limitations and translational gaps of the existing literature. Rather than providing confirmatory evidence, this work is intended as a hypothesis-generating framework to inform future epilepsy-focused studies evaluating whether modulation of neuroinflammatory and neurodegenerative pathways by CBG may hold relevance within disease-modifying research strategies for DRE.

## 1 Introduction

Drug-resistant epilepsy (DRE) affects approximately 30% of individuals with epilepsy and is defined by the failure to achieve sustained seizure freedom despite the appropriate use of at least two well-tolerated and adequately dosed antiseizure medications (ASMs). DRE is associated with a substantial clinical burden, including reduced quality of life, increased psychiatric comorbidity, progressive cognitive impairment, and a markedly elevated risk of sudden unexpected death in epilepsy (Kwan et al., 2010; Verducci et al., 2019). Increasing evidence indicates that, beyond insufficient symptomatic seizure control, persistent neuroinflammation, glutamatergic excitotoxicity, and progressive neurodegeneration contribute significantly to the pathophysiology and chronicity of DRE (Devinsky et al., 2013; Vezzani et al., 2019).

Within this evolving conceptual framework, interest has grown in neuromodulatory strategies that could engage inflammatory and neurodegenerative components of the disorder, alongside classical mechanisms of excitability control. Cannabidiol (CBD) represents the most clinically advanced phytocannabinoid in epilepsy, with established efficacy in specific DRE syndromes. Nevertheless, the recognition that DRE involves complex neuroimmune and neurodegenerative processes has motivated broader exploration of cannabinoid pharmacology beyond CBD, with particular attention to compounds that may engage multiple molecular pathways relevant to neuronal vulnerability and glial signaling (Aderinto et al., 2024; Anderson et al., 2021; Fleisher-Berkovich et al., 2023; Thiele et al., 2019).

Cannabigerol (CBG) is a non-psychoactive phytocannabinoid and a biosynthetic precursor of several major cannabinoids. Preclinical studies conducted across diverse experimental paradigms report that CBG can modulate inflammatory mediators, oxidative stress pathways, and cell-survival signaling (Borrelli et al., 2013; Kogan et al., 2021; Lah et al., 2021; Valdeolivas et al., 2015). Mechanistically, CBG has been reported to interact with cannabinoid receptors, transient receptor potential (TRP) channels, nuclear receptors such as peroxisome proliferator-activated receptor gamma (PPARγ), and additional neuromodulatory targets. Importantly, however, much of the available evidence derives from non-epilepsy models and *in vitro* systems, and the extent to which these mechanisms translate to seizure suppression, epileptogenesis, or network-level outcomes in epilepsy remains uncertain (Cascio et al., 2010; Echeverry et al., 2021; Granja et al., 2012; Iannotti et al., 2014; Lah et al., 2022; Lowin et al., 2023; Mendiguren et al., 2023).

Only a limited subset of experimental studies more directly addresses seizure-relevant contexts (e.g., excitotoxic or seizure-like paradigms and ion-channel mechanisms linked to neuronal hyperexcitability). These studies are essential for anchoring translational interpretation, but they also highlight a key limitation of the field: mechanistic effects on inflammation or neuronal survival do not necessarily imply antiseizure efficacy, and target engagement (such as voltage-gated sodium channel inhibition) may not, by itself, confer anticonvulsant effects. Accordingly, careful distinction between mechanistic plausibility and demonstrated epilepsy-specific outcomes is necessary.

In this review, we synthesize current preclinical evidence on CBG’s molecular targets and downstream pathways relevant to neuroinflammation, oxidative stress, and neuronal vulnerability, and we discuss how these mechanisms may intersect with biological processes implicated in DRE. Crucially, this review is intended as a hypothesis-generating framework rather than a confirmatory assessment of antiseizure efficacy. We aim to (i) clarify what is currently supported by the evidence, (ii) delineate translational limitations and sources of uncertainty, and (iii) identify concrete priorities for epilepsy-focused studies needed to determine whether CBG has meaningful relevance within disease-modifying strategies for DRE.

## 2 Neurobiology of drug-resistant epilepsy

Epilepsy affects more than 51.7 million individuals worldwide (95% UI 44.9–58.9 million), highlighting a substantial global health burden (Collaborators, 2025). ASMs remain first-line therapy. However, a significant proportion of patients do not achieve sustained seizure freedom. The International League Against Epilepsy defines DRE as the failure of adequate trials of two appropriately chosen and well-tolerated ASM regimens to produce sustained seizure freedom (Kwan et al., 2010). Beyond persistent seizures, DRE is increasingly conceptualized as a disorder involving maladaptive changes in brain networks. Accumulating evidence suggests progressive alterations in neuronal excitability, synaptic integration, and network connectivity that may evolve over time and contribute to treatment refractoriness (Bazhanova et al., 2021; Perucca et al., 2023). Importantly, these changes are not uniform across patients or epilepsy syndromes, underscoring the heterogeneity of the disorder and the complexity of its underlying biology.

At the neurobiological level, recurrent seizures have been associated with disruptions in inhibitory control and sustained increases in excitatory drive. Prolonged glutamatergic transmission may promote excitotoxic cascades capable of destabilizing neuronal circuits and increasing vulnerability to subsequent insults (Ramandi et al., 2021; Sumadewi et al., 2023; Vezzani et al., 2019). In parallel, chronic neuroinflammatory responses have been observed in both experimental models and human epileptic tissue. These responses commonly involve microglial activation, astrocytic reactivity, and altered cytokine and chemokine signaling—processes that can influence synaptic function, neuronal survival, and network stability over time (Feng et al., 2019; Li et al., 2023; Shi et al., 2025; Varvel et al., 2016). While such inflammatory changes are consistently associated with epilepsy and DRE, their precise causal contribution to seizure persistence and pharmacoresistance remains an area of active investigation.

Disruption of the blood–brain barrier (BBB) may further interact with excitotoxic and inflammatory processes associated with epilepsy. Experimental and clinical evidence suggests that BBB dysfunction can facilitate the entry of peripheral immune mediators and circulating immune cells into the brain parenchyma, thereby perturbing synaptic, metabolic, and vascular homeostasis (van Vliet et al., 2015). Such alterations have been linked to sustained network instability and maladaptive circuit reorganization, although their relative contribution likely varies according to disease stage, epilepsy subtype, and the temporal evolution of the disorder.

Taken together, excitotoxicity, neuroinflammatory signaling, and BBB impairment are increasingly regarded as interconnected processes associated with neuronal loss, circuit remodeling, and persistent network dysfunction in DRE. This integrative view supports a conceptual shift away from considering DRE as a static seizure phenotype and toward understanding it as a dynamic pathobiological state, in which progressive network alterations may contribute to reduced responsiveness to pharmacological treatment rather than arising from a single dominant mechanism (Aguilar-Castillo et al., 2025; Khambhati et al., 2021; Negi et al., 2023; Sun et al., 2023).

Consistent with this perspective, integrative analyses of DRE emphasize that pharmacoresistance cannot be adequately explained by any single hypothesis. Reviews such as that by Pérez-Pérez et al. (2021) highlight the limitations of individual explanatory models, including target alteration, transporter overexpression, network reorganization, and neuroinflammation, and argue for a global framework in which central and peripheral mechanisms converge to sustain treatment resistance. Importantly, this conceptual synthesis does not evaluate specific pharmacological classes or compounds, but rather underscores the need for hypothesis-driven approaches capable of addressing multiple interacting biological pathways.

## 3 Cannabigerol: molecular profile and pharmacodynamics

### 3.1 Chemical identity and biosynthetic origin

CBG (2-[(2E)-3,7-dimethylocta-2,6-dienyl]-5-pentylbenzene-1,3-diol) is a non-psychoactive terpenophenolic phytocannabinoid naturally occurring in *Cannabis sativa* L. Although historically less studied than CBD and THC, CBG has gained attention as a multi-target compound with potential relevance to neuroimmune modulation particularly within conceptual frameworks that emphasize pleiotropic pharmacology rather than single-target antiseizure mechanisms (Basavarajappa and Subbanna, 2024; Jastrząb et al., 2022; Li et al., 2024).

CBG is produced by decarboxylation of cannabigerolic acid (CBGA), a central biosynthetic intermediate from which major phytocannabinoid classes diverge (Gülck and Møller, 2020). CBGA arises from the condensation of geranyl diphosphate (GPP) with olivetolic acid via CBGA synthase (Xia et al., 2024). Enzymatic conversion by THCA-, CBDA-, or CBCA-synthases typically directs CBGA toward THC, CBD, or CBC derivatives. In the absence of these routes, or under thermal and photochemical conditions, CBGA can undergo non-enzymatic decarboxylation to yield CBG (Jastrząb et al., 2022; Manetto et al., 2024).

### 3.2 Physicochemical properties and target engagement

Structurally, CBG contains a resorcinol aromatic core substituted with a prenyl chain and a pentyl chain, conferring high lipophilicity (logP ≈7.1) (Luz-Veiga et al., 2024). This physicochemical profile may facilitate membrane permeation and interaction with hydrophobic binding pockets across G protein–coupled receptors (GPCRs), ion channels, and nuclear receptors (Jastrząb et al., 2022; Morales et al., 2017; O'Sullivan, 2016). However, high lipophilicity also complicates interpretation of *in vitro* potency, because effective concentrations may not directly translate to brain exposure *in vivo*. Accordingly, pharmacodynamic observations for CBG should be interpreted cautiously and, where possible, considered alongside available pharmacokinetic information, formulation-dependent effects, and route of administration. These factors are particularly relevant when extrapolating molecular interactions toward potential relevance in complex neurological disorders.

### 3.3 Cannabinoid receptors and endocannabinoid tone

**FIGURE 1:**
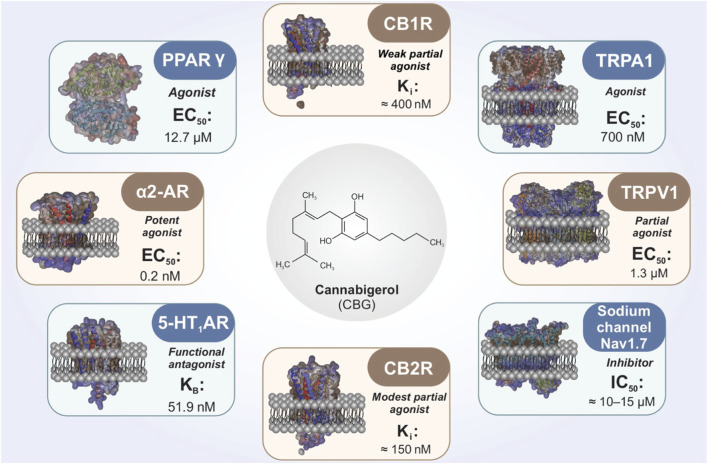
Molecular targets and pharmacodynamic profile of cannabigerol (CBG). This schematic summarizes the principal molecular targets reported for CBG, highlighting its pleiotropic pharmacodynamic profile. CBG has been described as an agonist of the nuclear receptor peroxisome proliferator-activated receptor gamma (PPARγ), a potent agonist at α_2_-adrenergic receptors (α_2_-AR), a functional antagonist at the 5-hydroxytryptamine 1A receptor (5-HT_1_A), and a partial agonist at transient receptor potential channels TRPV1 and TRPA1. In addition, CBG inhibits the voltage-gated sodium channel Nav1.7 and displays low-affinity binding with partial agonist activity at cannabinoid receptors CB1R and CB2R. Representative EC_50_, IC_50_, and Kᵢ values derived from preclinical studies are indicated to provide an approximate range of target engagement. Reported affinities and functional activities may vary depending on species, assay conditions, and experimental context.

CBG exhibits a pleiotropic pharmacodynamic profile (Figure 1) and engages diverse molecular targets that extend beyond classical cannabinoid receptor agonism (Jastrząb et al., 2022; Li et al., 2024). Within the ECS, CBG has been reported to act as a low-efficacy partial agonist at CB1R and CB2R receptors, with binding affinities in the low-to mid-nanomolar range (CB1R Ki ≈ 400 nM; CB2R Ki ≈ 150 nM) (Navarro et al., 2018; Pertwee, 2008). Its intrinsic efficacy is lower than THC, which is consistent with the absence of canonical CB1R-mediated psychoactive effects.

In addition to direct receptor interactions, CBG has been reported to influence endocannabinoid tone by modulating anandamide (AEA) handling and enzymes involved in endocannabinoid metabolism, including fatty acid amide hydrolase (FAAH) and monoacylglycerol lipase (MAGL) (Jarocka-Karpowicz et al., 2024). These mechanisms could, in principle, modulate neuronal excitability indirectly; however, whether such effects are sufficient to influence seizure generation, epileptogenesis, or pharmacoresistance *in vivo* remains uncertain and has not been directly demonstrated in epilepsy-specific models.

### 3.4 TRP channels relevant to excitability and neuroimmune signaling

Beyond cannabinoid receptors, CBG interacts with TRP channels implicated in nociception, neurogenic inflammation, and excitability. Reported activity includes partial agonism at TRPV1 (EC_50_ ≈ 1.3 μM), and agonism at TRPA1 (EC_50_ ≈ 700 nM) (De Petrocellis et al., 2011). TRPV1 activation can be followed by channel desensitization, a mechanism that may reduce excitatory signaling in certain settings. TRPA1 activation may modulate neurogenic inflammatory pathways and peripheral–central immune crosstalk (Muller et al., 2018).

Although modulation of TRP channels provides mechanistic plausibility for effects on excitability and inflammation, direct evidence linking CBG-mediated TRP channel activity to seizure suppression, network stabilization, or disease modification in DRE models remains limited.

### 3.5 PPARγ and inflammatory-metabolic signaling

A mechanistically distinct component of CBG pharmacology is its reported agonism at the PPARγ (EC_50_ ≈ 12.7 μM) (Granja et al., 2012). PPARγ activation is widely linked to suppression of Nuclear Factor kappa B (NF-κB) dependent pro-inflammatory signaling, enhancement of antioxidant responses (including Nrf2-associated pathways), and modulation of mitochondrial and metabolic programs (Senn et al., 2023). In microglia, PPARγ signaling is frequently associated with attenuation of pro-inflammatory activation states, a feature relevant to contemporary views of epileptogenesis and DRE that emphasize neuroimmune and metabolic contributions. Notably, most evidence linking PPARγ activation to beneficial outcomes derives from neuroinflammatory or neurodegenerative models rather than epilepsy-specific paradigms. Consequently, the extent to which CBG-driven PPARγ activity translates into disease-modifying effects in epilepsy remains an open question requiring targeted validation.

### 3.6 Additional neuromodulatory targets (5-HT1A, α2-AR, Nav channels)

CBG has also been reported to interact with additional neuromodulatory systems. Functional antagonism at serotonin 5-HT1A receptors (K_B_ ≈ 51.9 nM) has been discussed in relation to anxiety-related behaviors and neuroprotection in selected experimental paradigms (Cascio et al., 2010; Nachnani et al., 2021). CBG has further been described as a potent agonist at α_2_-adrenergic receptors (EC_50_ ≈ 0.2 nM), a property that could influence presynaptic neurotransmitter release and noradrenergic tone—processes implicated in seizure thresholds and network synchronization (Nachnani et al., 2021). CBG has also been reported to inhibit the voltage-gated sodium channel Na_v_1.7 (IC_50_ ≈ 10–15 μM), suggesting a potential role in modulating neuronal excitability (Ghovanloo et al., 2022). More broadly, recent work has demonstrated inhibitory effects of several lesser-studied phytocannabinoids on human voltage-gated sodium channels (Milligan et al., 2023). However, accumulating evidence indicates that sodium channel blockade by plant cannabinoids does not necessarily confer anticonvulsant efficacy (Hill et al., 2014). As such, the functional relevance of sodium channel modulation by CBG for seizure generation, network hyperexcitability, or pharmacoresistance in DRE remains unclear and represents a critical gap requiring epilepsy-specific investigation.

### 3.7 Translational note and interpretive cautions

Collectively, these molecular interactions support the view of CBG as a multi-target neuromodulator with potential relevance to inflammatory, metabolic, and excitability-related pathways implicated in DRE. Importantly, many of the anti-inflammatory and neuroprotective properties described for CBG overlap with those reported for CBD, and current evidence does not support claims of superiority of CBG over CBD in epilepsy. Moreover, a substantial portion of the mechanistic evidence summarized here derives from non-epilepsy disease models or *in vitro* systems that do not capture seizure generation, epileptogenesis, or network-level outcomes. Accordingly, this section should be interpreted as a pharmacological mapping exercise rather than an endorsement of CBG as an antiseizure therapy. At present, CBG is best positioned as a hypothesis-generating candidate whose molecular profile motivates targeted testing in epilepsy-relevant models and, ultimately, careful translational evaluation.

## 4 CBG and neuroinflammation: cellular and molecular targets

### 4.1 Anti-inflammatory and antioxidant effects in immune and glial cells

**FIGURE 2:**
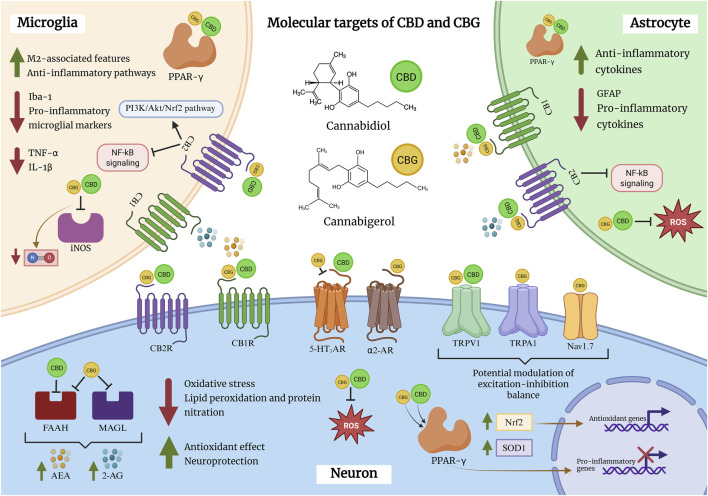
Molecular targets and proposed mechanisms of cannabidiol (CBD) and cannabigerol (CBG) in microglia, astrocytes, and neurons. This schematic summarizes reported molecular interactions through which CBD and CBG engage multiple targets, including cannabinoid receptors CB1 and CB2, transient receptor potential channels TRPV1 and TRPA1, the 5-HT1A receptor, α2-adrenergic receptors, and the nuclear receptor peroxisome proliferator-activated receptor gamma (PPAR-γ). In glial cells, preclinical studies suggest that both phytocannabinoids may modulate neuroinflammatory signaling, including attenuation of NF-κB–associated pathways, reduction of pro-inflammatory mediators, and promotion of anti-inflammatory responses, alongside decreased expression of markers associated with reactive astrocytic and microglial activation. In neurons, CBD and CBG have been reported to influence oxidative stress and redox homeostasis, potentially through Nrf2-related signaling and partial modulation of the endocannabinoid system via inhibition of the degradative enzymes FAAH and MAGL, which under certain conditions may increase levels of anandamide (AEA) and 2-arachidonoylglycerol (2-AG). Collectively, these mechanisms are proposed to contribute to the modulation of neuroinflammation, neuronal vulnerability, and excitability. Importantly, the depicted pathways are derived predominantly from preclinical studies, including *in vitro* and non-epilepsy *in vivo* models, and their relevance may vary depending on species, dose, and experimental context.

A growing body of preclinical evidence indicates that CBG exerts anti-inflammatory actions across immune, glial, and neuronal systems, primarily characterized in *in vitro* experimental settings. Studies in murine macrophages have shown that low micromolar concentrations of CBG attenuate lipopolysaccharide (LPS)-induced inflammatory responses, including reductions in nitrite accumulation and suppression of inducible nitric oxide synthase (iNOS) expression, consistent with inhibition of nitric oxide (NO)-dependent inflammatory signaling (Borrelli et al., 2013) (Figure 2).

Comparable effects have been reported in microglial models. In BV2 microglial cells, CBG decreases NO release and downregulates iNOS expression following LPS stimulation, with similar reductions observed in primary mixed glial cultures. Importantly, protein-level analyses indicate that CBG selectively suppresses stimulus-induced iNOS expression without altering basal levels, suggesting a context-dependent immunomodulatory profile rather than nonspecific immune suppression (Fleisher-Berkovich et al., 2023).

In addition to its anti-inflammatory actions, CBG attenuates hydrogen peroxide (H_2_O_2_)-induced oxidative stress in RAW 264.7 murine macrophages. This protective effect has been linked to CB2R signaling, as pharmacological blockade of CB2R abolishes the antioxidant effects of CBG, whereas CB1R inhibition does not. Under these conditions, CBG reduces oxidative stress markers such as iNOS and nitrotyrosine and increases superoxide dismutase 1 (SOD1) expression, supporting an antioxidant component to its immunomodulatory actions (Giacoppo et al., 2017) (Table 1).

**TABLE 1:** Preclinical evidence on the anti-inflammatory and neuroprotective effects of CBG. The table compiles *in vitro* and *in vivo* studies demonstrating that CBG reduces key pro-inflammatory mediators (e.g., iNOS, TNF-α, IL-1β, IL-6, IFN-γ, nitrotyrosine) and enhances anti-inflammatory or cytoprotective pathways (e.g., IL-10, IL-37, PPARγ, Nrf2). CBG also attenuates astrocytic and microglial activation markers (GFAP, Iba-1), highlighting its broad immunomodulatory actions across diverse inflammatory and neurodegenerative models.

Study type	Model	Cell line/Strain	CBG dose/Concentration	Results	References
*In vivo*	DNBS-induced colitis	Male ICR mice	30 mg/kg (post-injury)	↑IL-10 ↓IL-1β ↓INF-γ ↓iNOS	Borrelli et al. (2013)
*In vitro*	LPS-induced inflammation	Murine peritoneal macrophages	1 μM (pretreatment)	↓Nitrites ↓iNOS	
*In vivo*	3NP-induced Huntington’s disease (HD) model	Male C57BL/6 mice	10 mg/kg	↓TNF-α ↓IL-6 ↓iNOS ↓Iba-1 ↑GFAP	Valdeolivas et al. (2015)
*In vitro*	H₂O₂-induced oxidative stress	Murine RAW264.7 macrophages	10 μM	↓Nitrotyrosine ↓iNOS	Giacoppo et al. (2017)
*In vitro*	Neuroinflammation induced by conditioned medium from LPS-stimulated RAW macrophage cultures	Murine NSC-34 Motor Neurons	7.5 µM	↓TNF-α ↓IL-1β ↓IFN-γ ↓Nitrotyrosine ↓iNOS ↑Nrf-2	Gugliandolo et al. (2018)
*In vitro*	Neuroinflammation induced by conditioned medium from LPS-stimulated RAW 264.7 macrophage cultures	Murine NSC-34 Motor Neurons	2.5 µM, 5 µM	↑IL-10* ↑PPARγ ↑IL-37* ↑Nrf2* ↓TNF-α ↓NF-κB* ↓iNOS *with CBD 5 µM	Mammana et al. (2019)
*In vitro*	LPS-induced inflammation	Murine BV2 microglia	5 μM	↓NO ↓TNF-α ↓iNOS	Fleisher-Berkovich et al. (2023)
*In vivo*	MOG-induced Autoimmune Encephalomyelitis (EAE) model	Female C57BL/6 mice	10 mg/kg	↓GFAP	

### 4.2 Transcriptional regulation of inflammatory and antioxidant pathways

Beyond modulation of NO/iNOS signaling, CBG influences a broader inflammatory and oxidative stress–related transcriptome. In NSC-34 motor neurons exposed to inflammatory mediators derived from LPS-activated macrophages, CBG reduces the expression of pro-inflammatory cytokines, including tumor necrosis factor-α (TNF-α), interleukin-1β (IL-1β), interferon-γ (IFN-γ), and nitrotyrosine. Concomitantly, CBG upregulates PPARγ and nuclear factor erythroid 2–related factor 2 (Nrf2), two transcriptional regulators central to anti-inflammatory and antioxidant responses (Gugliandolo et al., 2018; Mammana et al., 2019).

Co-treatment with CBD further amplifies selected anti-inflammatory outcomes *in vitro*, including increased expression of interleukin-10 (IL-10) and interleukin-37 (IL-37). While these findings suggest complementary or potentially synergistic anti-inflammatory effects at the cellular level, their relevance to seizure modulation, epileptogenesis, or network-level outcomes in epilepsy has not been directly demonstrated and remains speculative at present.

### 4.3 *In vivo* modulation of glial reactivity

*In vivo* studies provide additional evidence that CBG can modulate glial reactivity within the CNS. In a murine model of neuroinflammation, CBG reduces expression of glial fibrillary acidic protein (GFAP) in the lumbar spinal cord, indicating attenuation of astrocytic activation (Fleisher-Berkovich et al., 2023). Similarly, in a Huntington’s disease model, CBG decreases the microglial activation marker ionized calcium-binding adaptor molecule 1 (Iba-1), consistent with suppression of microglial reactivity *in vivo* (Valdeolivas et al., 2015).

Although these experimental paradigms do not directly assess seizure activity, epileptogenesis, or pharmacoresistance, they provide proof-of-principle evidence that CBG can influence astrocytic and microglial responses under neuroinflammatory conditions. Such effects are relevant in light of the established contribution of glial activation to chronic neuroinflammation, network instability, and disease progression in DRE, but should not be interpreted as direct evidence of antiseizure efficacy.

### 4.4 CB2R signaling and microglial phenotype: inferred mechanisms and current limitations

To date, direct experimental demonstration of CBG-induced microglial phenotype switching from pro-inflammatory (M1-like) to anti-inflammatory (M2-like) states has not been reported. Consequently, the capacity of CBG to promote a neuroprotective microglial phenotype in the context of epilepsy remains largely inferential, extrapolated from mechanistic insights obtained in other neuroinflammatory disease models.

Substantial evidence indicates that CB2R signaling, a recognized molecular target of CBG, plays a central role in regulating microglial activation and polarization. In toxin-induced neuroinflammatory models, selective CB2R agonists such as JWH-133 suppress M1-associated features and promote expression of M2-related markers through PI3K/Akt/Nrf2-dependent pathways (Wang et al., 2023). Additional studies have demonstrated CB2R-driven microglial polarization via cAMP/PKA/CREB signaling in intracerebral hemorrhage models (Lin et al., 2017), with similar immunoregulatory trends reported in Alzheimer’s disease (AD) paradigms (Ferrisi et al., 2023).

Importantly, epilepsy-relevant *in vitro* seizure models have shown that phytocannabinoids such as CBD attenuate kainate-induced neuronal damage and microglial activation (Landucci et al., 2022), providing a mechanistic framework linking cannabinoid-mediated immunomodulation to excitotoxic injury. Within this context, the anti-inflammatory profile of CBG is mechanistically consistent with established CB2R-dependent pathways. Nevertheless, current evidence supports an inferred rather than directly demonstrated role for CBG in modulating microglial phenotype in epilepsy. This distinction is critical, as extrapolation from CB2R agonist studies or non-epileptic disease models cannot substitute for direct experimental validation.

Accordingly, future studies employing epilepsy-relevant models, cell-type–specific approaches, and longitudinal designs will be required to determine whether CBG can actively promote M2-like, neuroprotective microglial states and whether such effects meaningfully contribute to disease modification in DRE.

## 5 CBG in neurodegeneration and neuronal survival

Available preclinical evidence indicates that CBG exerts neuroprotective effects across a range of experimental models characterized by metabolic stress, neuroinflammation, excitotoxicity, and progressive neuronal loss (Table 2). *In vivo*, CBG administration (10 mg·kg^−1^) attenuates striatal neurodegeneration induced by 3-nitropropionic acid (3-NP), a mitochondrial complex II inhibitor widely used to model Huntington-like pathology. Under these conditions, CBG preserves neuronal architecture and supports partial recovery of striatal neuron populations, as demonstrated by Nissl staining and NeuN immunoreactivity (Valdeolivas et al., 2015).

**TABLE 2:** Preclinical evidence of CBG’s neuroprotective actions. The table summarizes *in vitro* and *in vivo* studies showing that CBG enhances neuronal survival and plasticity across multiple models of neurodegeneration and neuroinflammation.

Study type	Model	Cell line/Strain	CBG dose/Concentration	Results	References
*In vivo*	3NP-induced Huntington’s disease (HD) model	Male C57BL/6 mice	CBG (10 mg/kg)	↑Neural architecture ↑NeuN	Valdeolivas et al. (2015)
*In vitro*	Neuroinflammation induced by conditioned medium from LPS-stimulated RAW 264.7 macrophage cultures	Murine NSC-34 Motor Neurons	CBG (5 µM)	↑Cell viability ↓Bax	Mammana et al. (2019)
*In vitro*	TBHP-induced neurotoxicity	Rat PC12 pheochromocytoma cells	CBG (1 μM)	↑Cell viability	Marsh et al. (2024)
	Aβ1–42-induced Alzheimer’s disease (AD) model			↑Neuroprotection ↑Neurite outgrowth	
*In vivo*	MOG-induced Autoimmune Encephalomyelitis (EAE) model	Female C57BL/6 mice	CBG (10 mg/kg)	↓Neuronal loss	Fleisher-Berkovich et al. (2023)
*In vivo*	Bilateral Common Carotid Arteries Occlusion (BCCAO)	Male C57BL/6 mice	CBG (1, 5, 10 mg/kg)	↓Neuronal loss ↑NeuN ↑MAP2	Kohara et al.(2025)

Complementary *in vitro* studies reinforce this neuroprotective profile. In NSC-34 motor neurons exposed to inflammatory mediators released from LPS-activated RAW264.7 macrophages, CBG at micromolar concentrations (5 μM) enhances cell viability and attenuates neurotoxic signaling (Mammana et al., 2019). Together, these findings support a role for CBG in preserving neuronal integrity under conditions of inflammation-driven secondary injury, rather than indicating direct effects on seizure generation or excitability.

### 5.1 Modulation of apoptotic and oxidative stress pathways

Within the same experimental framework, co-administration of CBG and CBD at equimolar concentrations (5 μM) further reduced markers of apoptotic signaling. Specifically, combined treatment suppressed NF-κB activation, decreased expression of the pro-apoptotic protein Bax, and increased levels of the anti-apoptotic regulator Bcl-2 (Mammana et al., 2019). These results suggest complementary actions of the two non-psychoactive cannabinoids at the cellular level.

However, these observations derive exclusively from *in vitro* paradigms and should be interpreted with caution. At present, there is no direct evidence that such combinatorial effects translate into improved seizure control, modulation of epileptiform activity, or stabilization of hyperexcitable networks in epilepsy models.

Additional support for CBG-mediated neuroprotection comes from studies using PC12 cells exposed to combined oxidative and amyloidogenic stressors (tert-butyl hydroperoxide and Aβ_1-42_). Under these conditions, CBG at low micromolar concentrations (1 μM) sustains neuronal viability and promotes a phenotype consistent with reduced oxidative burden (Marsh et al., 2024). Collectively, these findings implicate attenuation of oxidative stress and apoptotic cascades as central components of CBG’s neuroprotective actions across diverse experimental contexts.

### 5.2 Neuroprotection in ischemic injury models

Recent evidence further indicates that CBG can enhance neuronal survival in models of acute ischemic injury. In a transient global cerebral ischemia model induced by bilateral common carotid artery occlusion (BCCAO) in C57BL/6 mice, intraperitoneal administration of CBG (1, 5, or 10 mg·kg^−1^) for 7 days beginning 1 hour after occlusion significantly attenuated ischemia-induced memory deficits and reduced hippocampal neuronal loss (Kohara et al., 2025).

Histopathological analyses revealed preservation of neuronal density within the CA1 and CA3 hippocampal subfields, accompanied by increased expression of proteins associated with synaptic plasticity. These neuroprotective effects occurred alongside reduced microglial and astrocytic reactivity, as indicated by decreased expression of Iba-1 and GFAP, as well as modulation of inflammatory markers. Together, these findings suggest that CBG may promote neuronal survival through coordinated suppression of neuroinflammation and support of plasticity-related repair mechanisms in ischemic contexts.

### 5.3 Relevance to epilepsy and interpretive limitations

Although the neuroprotective effects of CBG appear consistent across multiple experimental paradigms, it is important to emphasize that the majority of available evidence derives from non-epileptic disease models or *in vitro* systems. While oxidative stress, neuroinflammation, mitochondrial dysfunction, and apoptotic signaling are increasingly recognized as contributors to epileptogenesis and the progression of DRE, these models do not directly assess seizure generation, network hyperexcitability, or pharmacoresistance.

Accordingly, current data support a mechanistic rationale for considering CBG as a modulator of neurodegenerative and neuronal survival–related pathways that may be relevant to the broader pathobiology of DRE, rather than as evidence of established disease-modifying or antiseizure efficacy. Targeted studies employing epilepsy-specific models, longitudinal designs, and network-level outcome measures will be required to determine whether CBG-mediated neuroprotection translates into meaningful effects on seizure burden, circuit remodeling, or long-term treatment responsiveness.

## 6 Therapeutic insights and future directions

Recent preclinical research has highlighted CBG as a multi-target neuromodulatory compound with potential relevance to the complex pathobiology of DRE. Importantly, the available evidence does not support positioning CBG as a replacement for established therapies such as CBD, whose antiseizure efficacy and clinical utility are supported by randomized controlled trials in specific epilepsy syndromes. Rather than functioning as a conventional antiseizure medication, CBG should be considered within an exploratory framework focused on mechanisms that may influence disease progression and network pathology beyond direct seizure suppression.

CBG exhibits a pleiotropic pharmacological profile, interacting with cannabinoid receptors, adrenergic and serotonergic systems, TRP channels, and nuclear receptors such as PPARγ. Through these interactions, CBG has been shown to modulate neuroinflammatory signaling, oxidative stress responses, apoptotic pathways, and aspects of synaptic function in a variety of experimental systems. However, the relative contribution of each molecular target to epilepsy-relevant outcomes remains poorly defined, and the extent to which these mechanisms converge to influence seizure generation, network hyperexcitability, or pharmacoresistance has not been established experimentally.

A central insight emerging from recent studies is the potential relevance of neuroimmune modulation to the pathophysiology of DRE. Sustained activation of microglia and astrocytes, dysregulated cytokine signaling, and BBB dysfunction are increasingly recognized as contributors to epileptogenesis, network instability, and reduced responsiveness to ASMs. In this context, preclinical data demonstrate that CBG attenuates lipopolysaccharide-induced inflammatory responses in immune and glial cells, including reductions in NO, TNF-α, and iNOS expression (Fleisher-Berkovich et al., 2023).

Beyond suppression of classical inflammatory mediators, CBG engages transcriptional regulators such as PPARγ and Nrf2, thereby linking immunomodulatory effects to metabolic and antioxidant pathways. These mechanisms are largely absent from conventional ASMs and provide a biologically plausible rationale for investigating CBG within disease-modifying research strategies. Nevertheless, it must be emphasized that most supporting evidence derives from non-epilepsy disease models or *in vitro* systems, underscoring the need for cautious interpretation and epilepsy-specific validation.

### 6.1 Combination strategies: opportunities and limitations

The pleiotropic pharmacology of CBG raises the possibility of combination strategies aimed at targeting multiple pathological processes simultaneously. *In vitro* studies indicate that co-administration of CBG and cannabidiol CBD can produce complementary anti-inflammatory and anti-apoptotic effects, including suppression of NF-κB signaling, reduced NO production, and modulation of apoptotic regulators such as Bax and Bcl-2 (Mammana et al., 2019; Marsh et al., 2024). These findings suggest the potential for additive or context-dependent synergistic interactions under controlled experimental conditions.

Importantly, recent work has extended this exploration into an epilepsy-relevant paradigm. Zhou et al. (2026), systematically evaluated the anticonvulsant effects of CBD and CBG, administered both individually and in combination, in the murine maximal electroshock (MES) seizure model. Both phytocannabinoids demonstrated comparable efficacy, with ED_50_ values of approximately 133 mg/kg for CBD and 149 mg/kg for CBG. Notably, co-administration at a 1:1 ratio reduced the combined ED_50_ to approximately 60 mg/kg and was associated with an increased therapeutic index (∼5.3), despite a concomitant reduction in TD_50_. Isobolographic analysis indicated a predominantly additive interaction, consistent with functional convergence of anticonvulsant mechanisms rather than supra-additive potentiation. These data provide quantitative evidence that CBG exhibits intrinsic anticonvulsant activity in an acute seizure paradigm and may influence the efficacy-toxicity relationship of CBD in a validated model of generalized tonic-clonic seizures.

Nevertheless, several limitations temper the translational interpretation of these findings. The MES model reflects acute seizure suppression and does not address pharmacoresistance, epileptogenesis, or long-term network remodeling. Accordingly, while the study provides meaningful epilepsy-relevant data, it does not establish disease-modifying efficacy or confirm therapeutic utility in DRE. Similar caution applies to other combination strategies. For example, additive anti-inflammatory effects have been reported for CBG in combination with telmisartan in microglial systems; however, these observations remain confined to *in vitro* neuroinflammatory models and have not been evaluated in epilepsy-specific contexts (Fleisher-Berkovich et al., 2023).

Despite growing mechanistic interest, substantial translational gaps remain before CBG can be meaningfully evaluated within the therapeutic framework of DRE. Key priorities include rigorous pharmacokinetic and dose-finding studies, characterization of brain penetration and target engagement, and systematic evaluation of safety and tolerability profiles both alone and in combination with existing therapies. In contrast to CBD, whose clinical use is informed by well-documented drug-drug interactions, interindividual variability, and dose-dependent adverse effects, the pharmacokinetic behavior, interaction potential, and tolerability profile of CBG in humans remain insufficiently characterized.

Future research should prioritize epilepsy-relevant experimental models capable of assessing seizure burden, epileptogenesis, network remodeling, and long-term treatment responsiveness. Clarifying whether CBG-mediated modulation of neuroinflammation and neuronal survival translates into clinically meaningful outcomes will be essential for defining its potential role, if any, within the therapeutic landscape of drug-resistant epilepsy.

## 7 Conclusion

Recent preclinical research has highlighted CBG as a multi-target phytocannabinoid capable of modulating neuroinflammatory, oxidative, and cell-survival pathways across diverse experimental systems. These biological processes are increasingly recognized as contributors to epileptogenesis and disease progression; however, it is important to emphasize that most available evidence derives from non-epilepsy models or *in vitro* studies. Consequently, direct support for antiseizure efficacy or disease modification by CBG in epilepsy remains limited.

Accordingly, CBG should be regarded as a hypothesis-generating candidate rather than an established antiseizure therapy. Its pleiotropic pharmacological profile justifies rigorous evaluation in epilepsy-relevant models that directly assess network hyperexcitability, pharmacoresistance, neuroimmune dynamics, and long-term neuronal outcomes, while carefully distinguishing ion-channel effects from functional anticonvulsant efficacy. Clarifying pharmacokinetics, safety, and potential interactions with existing antiseizure medications will be essential to determine whether CBG can meaningfully contribute to future disease-modifying strategies for drug-resistant epilepsy.
